# Lipopolysaccharides-Induced Suppression of Innate-Like B Cell Apoptosis Is Enhanced by CpG Oligodeoxynucleotide and Requires Toll-Like Receptors 2 and 4

**DOI:** 10.1371/journal.pone.0165862

**Published:** 2016-11-03

**Authors:** Xiaoqian Yu, Yuhua Wang, Jiang Lin, Yang Hu, Toshihisa Kawai, Martin A. Taubman, Xiaozhe Han

**Affiliations:** 1 The Forsyth Institute, Department of Immunology and Infectious Diseases, Cambridge, MA, United States of America; 2 Peking University School and Hospital of Stomatology, Department of Periodontology, Beijing, China; 3 Ninth People’s Hospital, College of Stomatology, Shanghai JiaoTong University School of Medicine, Department of Prosthodontics, Shanghai Key laboratory, Shanghai, China; 4 The Fourth Hospital of Harbin Medical University, Department of stomatology, Harbin, China; Temple University School of Medicine, UNITED STATES

## Abstract

Innate-like B lymphocytes play an important role in innate immunity in periodontal disease through Toll-like receptor (TLR) signaling. However, it is unknown how innate-like B cell apoptosis is affected by the periodontal infection-associated innate signals. This study is to determine the effects of two major TLR ligands, lipopolysaccharide (LPS) and CpG-oligodeoxynucleotides (CpG-ODN), on innate-like B cell apoptosis. Spleen B cells were isolated from wild type (WT), TLR2 knockout (KO) and TLR4 KO mice and cultured with *E*. *coli* LPS alone, *P*. *gingivalis* LPS alone, or combined with CpG-ODN for 2 days. B cell apoptosis and expressions of specific apoptosis-related genes were analyzed by flow cytometry and real-time PCR respectively. *P*. *gingivalis* LPS, but not *E*. *coli* LPS, reduced the percentage of AnnexinV^+^/7-AAD^-^ cells within IgM^high^CD23^low^CD43^-^CD93^-^ marginal zone (MZ) B cell sub-population and IgM^high^CD23^low^CD43^+^CD93^+^ innate response activator (IRA) B cell sub-population in WT but not TLR2KO or TLR4KO mice. CpG-ODN combined with *P*. *gingivalis* LPS further reduced the percentage of AnnexinV^+^/7-AAD^-^ cells within MZ B cells and IRA B cells in WT but not TLR2 KO or TLR4 KO mice. Pro-apoptotic CASP4, CASP9 and Dapk1 were significantly down-regulated in *P*. *gingivalis* LPS- and CpG-ODN-treated B cells from WT but not TLR2 KO or TLR4 KO mice. Anti-apoptotic IL-10 was significantly up-regulated in *P*. *gingivalis* LPS- and CpG-ODN-treated B cells from WT and TLR2 KO but not TLR4 KO mice. These results suggested that both TLR2 and TLR4 signaling are required for *P*. *gingivalis* LPS-induced, CpG-ODN-enhanced suppression of innate-like B cell apoptosis.

## Introduction

Innate immune system recognizes pathogen-associated molecular patterns with a set of germline-encoded pattern-recognition receptors including Toll-like receptors (TLRs) [1, 2]. TLRs play important roles in the process of B cell proliferation and apoptosis, and studies have shown that TLR2, TLR4 and TLR9 are all expressed in murine B cells [3, 4] as well as in human B cells [5, 6]. As multiple TLRs could be activated simultaneously by their corresponding ligands during immune response to pathogens in diseases, the effect of co-activation of these TLR pathways on B cell apoptosis has not been investigated.

Periodontal disease is an infection-associated, immune-mediated oral disease leading to the gingival tissue destruction [7], alveolar bone resorption [8], and increased risk of systemic complications [9]. *Porphyromonas gingivalis* (*P*. *gingivalis*), an anaerobic bacterium, is considered one of the principal pathogens of adult periodontitis that can orchestrate inflammatory disease by remodeling a normally benign microbiota into a dysbiotic one [10]. Different from *E*. *coli* LPS, which is a definitive TLR4 ligand, *P*. *gingivalis* LPS has been shown to be able to activate both TLR2 and TLR4 [11, 12]. Together with the ligation between bacterial DNA component CpG oligodeoxynucleotides (CpG-ODN) and its receptor TLR9 during *P*. *gingivalis* infection, it is valuable to determine the effects of multiple TLR activation (TLR2, TLR4 and TLR9) in the regulation of immune B cell functions in order to understand the role of TLR signaling in infection-associated periodontal pathogenesis.

B cells are linked developmentally, reside in different regions in the lymphoid organs, and mediate distinct functions [13]. In mice, three major B subsets have been identified as follicular B2 cells, B1 cells (including CD51B1a and CD52 B1b cells) and marginal zone (MZ) B cells. Innate-like B cells are heterogeneous populations that can rapidly acquire immune regulatory activities through the secretion of natural IgM and IL-10 [14]. These unconventional B cells with autoreactive properties can provide a rapid T cell-independent antibody response to protect against infections [15]. Innate-like B cells in mice are composed of B1 cells [16], marginal zone (MZ) B cells [17] and other related B cells [18]. Recent studies indicated that innate-like B cells can link innate immunity to adaptive immune responses during infection [19, 20].

Programmed cell death, including apoptosis, autophagy and programmed necrosis, is mediated by intracellular programs to decide the fate of cells [21]. Among the three forms of programmed cell death, apoptosis is a major event during immune cell development and responses to extracellular stimuli. Regulation of immune cell apoptosis is essential for the maintenance of immune system homeostasis [22, 23], and dysregulation of apoptosis in B cells may cause autoimmune manifestations [24]. Although numerous studies have indicated the key role of TLR signaling in the regulation of non-immune cell apoptosis [25, 26], the potential role of multiple TLRs in the control of innate-like B cell apoptosis is completely unknown.

The purpose of the study is to evaluate the role of specific TLRs on the innate-like B cell apoptosis using periodontal pathogen-associated TLR ligands (*P*. *gingivalis* LPS and CpG-ODN). Information on the TLR-mediated control of innate-like B cell apoptosis will give a new insight of host-pathogen interactions in the development of host immune response and periodontal disease pathogenesis.

## Materials and Methods

### Animals

C57BL/6 mice were purchased from the Jackson Laboratory (Bar Harbor, ME). TLR2 knockout (KO) and TLR4 KO mice backcrossed to the C57BL/6 background were a kind gift from Dr. Toshihisa Kawai (Forsyth Institute, Cambridge, USA). All the mice used in the study were from 8 to 10 weeks old and were maintained under pathogen-free conditions in laminar flow cabinets. The experimental protocols were approved by the Institutional Animal Care and Use Committee of the Forsyth Institute.

### B cell isolation and culture

Mice were euthanized in CO_2_ chamber and spleens were harvested. All cell culture disposable ware, including tips, tubes, serological pipettes, flasks and culture plates, were purchased from USA Scientific, Inc and were RNase, DNase, DNA, and pyrogen free. Designated biological safety cabinet and work area were used for culture experiments and were stringently cleaned and disinfected at all times. To monitor potential LPS contamination, the presence of bacterial endotoxins in buffers and culture medium were routinely performed by limulus amebocyte lysate (LAL) test using chromogenic endotoxin quantitation kit (Thermo Scientific). Splenic cell suspensions were prepared in MACS buffer (PBS/2mM EDTA/0.5% BSA). Non-B cells were depleted by incubating splenic cell suspensions with biotin-conjugated antibodies against CD4, CD11c, CD49b, CD90, Gr-1, and Ter119, followed by incubation with anti-biotin antibodies coupled magnetic beads (Miltenyi Biotec). Unlabeled cells were collected by magnetic depletion of labeled cells (contained >98.5% CD19+ cells). Isolated B cells were adjusted to 1×10^6^/ml and were added into either 96-well plates (200μl/well) in IMDM complete medium containing 10% FCS, 100 U/ml penicillin, 100 mg/ml streptomycin, 2 mM L-glutamine, 2.5μg/ml Amphotericin B (Hyclone, Thermo Fisher Scientific, IL) and 50 μM 2-ME. Cells were cultured at 37°C in a humidified incubator with 5% CO_2_. The TLR ligands were added to the B cells culture as follows: *E*. *coli* LPS (10μg/ml, strain O55:B5, Sigma-Aldrich), *P*. *gingivalis* LPS (10μg/ml, strain ATCC 33277, InvivoGen) and mouse stimulatory CpG-ODN (10μM, 5’-TCGTCGTTTTGTCGTTTTGTCGTT-3’, Hycult Biotech).

### B cell proliferation analysis

B cells (2×10^5^/well) were cultured in 200μl complete medium in 96-well plate for 2 days in the presence of *E*. *coli* LPS (10μg/ml), *P*. *gingivalis* LPS (10μg/ml), *E*. *coli* LPS (10μg/ml) + CpG (10μM), or *P*. *gingivalis* LPS (10μg/ml) + CpG (10μM). To determine the number of viable cells in proliferation, MTS reagent was added (40μl/well) 4 hours before the termination of the experiment using a CellTiter 96 AQueous Assay kit (Promega Corp). After 4 hour incubation, the plate was read at OD 490nm using a microplate reader (BioTek). The absorbance of the formazan at 490nm was measured as an indication of cell proliferation. Cell proliferation was also measured by CellTrace Cell Proliferation kit (Invitrogen) following manufacture instructions. Briefly, cells were stained with CellTrace CFSE reagents for 20 minutes and then incubated in culture medium for 10 minutes to undergo acetate hydrolysis. Proliferated cell were analyzed by flow cytometry at 488 nm excitation wavelength and at least 20,000 cells were counted for each sample.

### B cell apoptosis analysis by flow cytometry

Isolated B cells (1×10^6^/well) in 200μl culture medium were cultured for 48 hours in “U” bottom 96-well plate with *E*. *coli* LPS (10μg/ml), *P*. *gingivalis* LPS (10μg/ml), *E*. *coli* LPS (10μg/ml) + CpG (10μM), or *P*. *gingivalis* LPS (10μg/ml) + CpG (10μM). At the termination of cell culture, B cells in the 96-well plates were washed with PBS followed by incubation with fluorescence conjugated antibodies. The following anti-mouse monoclonal antibodies (mAbs) were used in subpopulation analysis to distinguish cells: PE-conjugated anti IgM, PerCP-Cy5.5-conjugated anti CD23, APC-conjugated anti CD93, Pacific Blue-conjugated anti CD43 (BD Biosciences). The following mAbs were also used for the analysis of B cell apoptosis: FITC- or PE-conjugated Annexin V (BD), 7-Aminoactinomycin D (7-AAD) (BioLegend). Annexin V^+^7-AAD^-^ cells are considered as early apoptotic cells and Annexin V^+^7-AAD^+^ cells are considered as late apoptotic cells. At least 50,000 cells were counted for each sample.

### Apoptosis-related gene array

Total RNA was extracted from cultured B cells using a Purelink RNA mini kit (Invitrogen). The mouse RT^2^ ProfilerPCR array for Apoptosis (PAMM-012Z, SA Biosciences) were used to profile expression of 84 apoptosis-related genes involved in programmed cell death, using a Roche real-time PCR machine (Roche Diagnostics Corporation, Indianapolis, IN). The data for biological duplicates were analyzed using the PCR Array Data Analysis Software (SABiosciences).

### Real-time PCR

Total RNA was extracted from the cultured B cells using a Purelink RNA mini kit (Life Technology, Carlsbad, CA) following manufacturer’s instructions. Isolated mRNA (0.1μg each) was reverse transcribed into cDNA using the SuperScriptII reverse transcription system in the presence of random primers (Invitrogen). The real-time PCR was carried out in a 20μl reaction system using SuperScript II Platinum SYBR Green Two-Step qRT-PCR Kit (Life Technology) in a Roche LightCycler 480 (Roche Diagnostics, Indianapolis, IN). Each cDNA sample was loaded in duplicate into the plate with a template amount of 10ng. The primers used for specific genes analyzed were from RT² qPCR Primer Assays (SA Biosciences). The real-time PCR conditions were: 95°C for 10 minutes, followed by 40 cycles of 95°C for 10seconds, 65°C for 10 seconds and 72°C for 15 seconds. Results were presented as fold changes relative to GAPDH reference.

### Casp4 and Casp9 activity assay

Splenocyte B cells were separated from WT, TLR2KO and TLR4KO mice and cultured 48 hours with *P*. *gingivalis* LPS (10μg/ml), *P*. *gingivalis* LPS (10μg/ml) + CpG (10μM) and untreated control. Casp4 and Casp9 protein activities were performed by using Caspase 4 Assay kit (Abcam) or Caspase 9 Assay kit (Abcam) following user’s instruction. Briefly, cells (1×10^6^ per sample) were lysis in 50 μl cell lysis buffer incubated on ice for 10 minutes and then incubated with 50 μl reaction buffer and 5 μl LEHD-AFC substrate at 37°C for 2 hours. The plate was read in a microplate fluorometer reader (BioTek) and fold-increase in Caspase 4/9 activity was determined by comparing these results with the level of the untreated control.

### Statistics

Results are presented as means ± standard errors (SE). Paired Student’s t-test was used to analyze differences between two treatments. One-Way ANOVA was used to analyze differences among groups. Results with probability values of less than 0.05 are considered statistically significant.

## Results

### B cell proliferation after treatment with LPS and CpG-ODN

**Fig 1:**
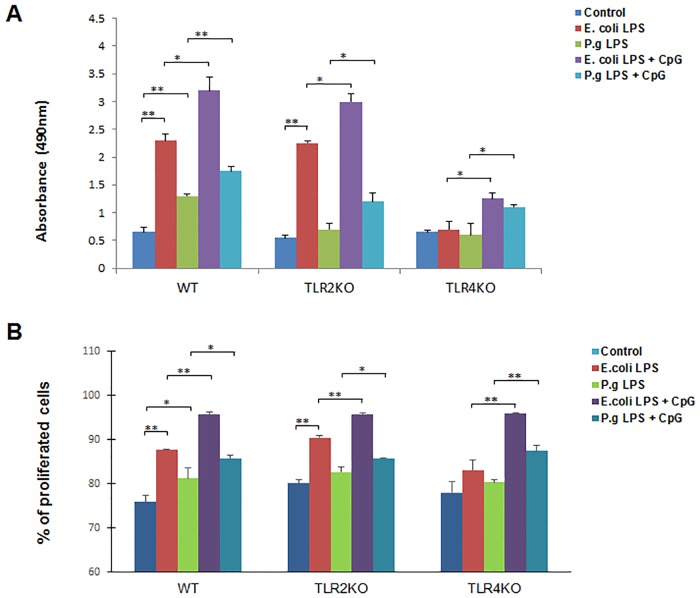
B cell proliferation after *E*. *coli* LPS, *P*. *gingivalis* LPS and CpG-ODN treatment. Splenocyte B cells were separated from WT, TLR2 KO and TLR4 KO mice and cultured 48 hours with *E*. *coli* LPS (10μg/ml), *P*. *gingivalis* LPS (10μg/ml), *E*. *coli* LPS (10μg/ml) + CpG (10μM), and *P*. *gingivalis* LPS (10μg/ml) + CpG (10μM). Viable cells quantities were measured by absorbance at 490 nm reading from each group of WT, TLR2 KO and TLR4 KO mice respectively (**A**) (mean±SE, n = 6, *p<0.05, **p<0.01). Proliferation cells quantities were also measured by CellTrace CSFE staining and presented as percentages of total cells (**B**). (mean±SE, n = 3, *p<0.05, **p<0.01).

To test the innate proliferative property of B cells in response to the TLRs stimulation, purified B cells were cultured under 5 different conditions (untreated control, *E*. *coli* LPS, *P*. *gingivalis* LPS, *E*. *coli* LPS + CpG-ODN and *P*. *gingivalis* LPS + CpG-ODN) and cell proliferation assays were performed after 48 hours. *E*. *coli* LPS strongly stimulated the proliferation of B cells from WT and TLR2 KO mice (Fig 1A, 2^nd^ bar in each type of animal). *P*. *gingivalis* LPS stimulated proliferation of B cells from WT mice only (Fig 1A, 3^rd^ bar in each type of animal), and the intensity of such stimulation was weaker than those observed in *E*. *coli* LPS. In all types of mice, the addition of CpG-ODN together with LPS significantly elevated the proliferation of B cells as compared to those treated with LPS alone (Fig 1A, 4^th^ and 5^th^ bars in each group of animal). To confirm the MTS proliferation results, cell proliferations of each groups were also measured by CellTrace CSFE cell proliferation assay and similar results were observed, demonstrating that the addition of CpG-ODN together with LPS significantly elevated the proliferation of B cells as compared to those treated with LPS alone (Fig 1B).

### Inhibition of B cell early- and late-apoptosis by *P*. *gingivalis* LPS and CpG-ODN

**Fig 2:**
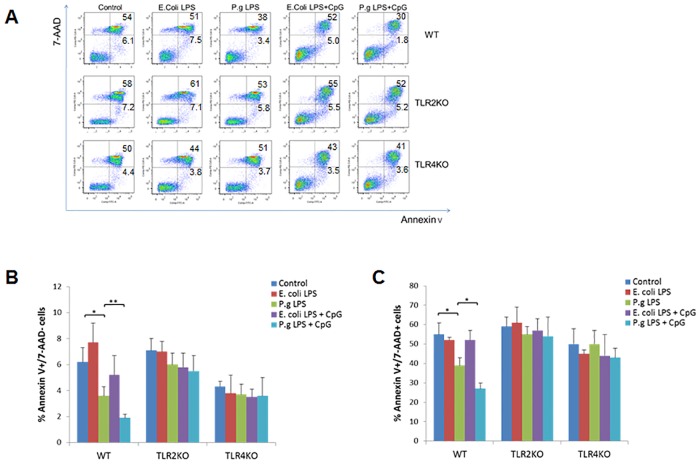
B cell early apoptosis and late apoptosis after *E*. *coli* LPS, *P*. *gingivalis* LPS and CpG-ODN treatment. Splenocyte B cells were separated from WT, TLR2 KO and TLR4 KO mice and cultured 48 hours with *E*. *coli* LPS (10μg/ml), *P*. *gingivalis* LPS (10μg/ml), *E*. *coli* LPS (10μg/ml) + CpG (10μM), and *P*. *gingivalis* LPS (10μg/ml) + CpG (10μM). Cells were then stained by FITC-conjugated AnnexinV mAb and 7-AAD and measured by flow cytometry (**A**). Percentage of Annexin V^+^7-AAD^-^ cells (**B**) and Annexin V^+^7-AAD^+^ cells (**C**) in different treatment groups of WT, TLR2KO and TLR4KO mice were analyzed and compared respectively (mean±SE, n = 6, *p<0.05, **p<0.01).

To determine the overall B cell apoptosis, purified B cells from WT and TLRs KO mice were cultured for 2 days under different treatment conditions followed by staining with AnnexinV and 7-AAD and analyzed by flow cytometry (Fig 2A). In WT mice, the percentage of AnnexinV^+^/7-AAD^-^ (early apoptotic) B cells was significantly decreased after treatment with *P*. *gingivalis* LPS (*p*<0.05) as compared to control group (Fig 2B). The percentage of AnnexinV^+^/7-AAD^-^ B cells was further reduced when treated with *P*. *gingivalis* LPS and CpG-ODN (*p*<0.01) as compared to group treated with *P*. *gingivalis* LPS alone (Fig 2B). However, the percentage of AnnexinV^+^/7-AAD^-^ B cells was not changed after treatment with *E*. *coli* LPS alone, or combined with CpG-ODN, when compared to their respective controls. No changes were observed in the percentage of AnnexinV^+^/7-AAD^-^ B cells from TLR2 KO or TLR4 KO mice under each treatment condition (Fig 2B). Similar results were observed when the percentage of AnnexinV^+^/7-AAD^+^ (late apoptotic/necrotic) B cells was evaluated after different treatments. Only in WT mice, the percentage of AnnexinV^+^/7-AAD^+^ B cells was significantly decreased after treatment with *P*. *gingivalis* LPS (*p*<0.05), but not with *E*. *coli* LPS, and such effect was further enhanced by the addition of CpG-ODN (*p*<0.05) (Fig 2C).

### Different responses of innate-like B cell subsets after treatment with LPS and CpG-ODN

**Fig 3:**
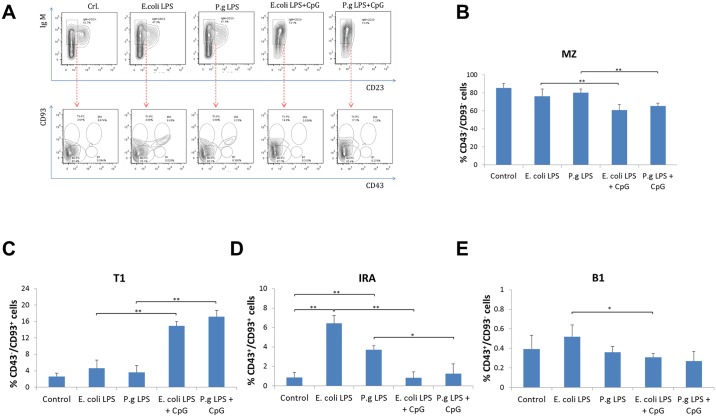
Frequencies of Innate-like B cell subsets after *E*. *coli* LPS, *P*. *gingivalis* LPS and CpG-ODN treatment. Splenocyte B cells were separated from WT mice and cultured 48 hours with *E*. *coli* LPS (10μg/ml), *P*. *gingivalis* LPS (10μg/ml), *E*. *coli* LPS (10μg/ml) + CpG (10μM), and *P*. *gingivalis* LPS (10μg/ml) + CpG (10μM). Cells were then stained by PE-conjugated anti IgM, PerCP-Cy5.5-conjugated anti CD23, APC-conjugated anti CD93, Pacific Blue-conjugated anti CD43 and measured by flow cytometry (**A**). Within overall innate-like B cell (IgM^high^CD23^low^ B cells) population, the percentage of CD43^-^CD93^-^ marginal zone B cells (**B**), CD43^-^CD93^+^ transitional B cells (**C**), CD43^+^CD93^+^ innate response activator B cells (**D**) and CD43^+^CD93^-^ B1 B cells (**E**) in different treatment groups were analyzed and compared respectively (mean±SE, n = 4, *p<0.05, **p<0.01).

The percentage of four sub-types of innate-like B cells in WT mice were detected by flow cytometry using surface markers as previously described [18] to evaluate the innate-like B cell responses to LPS and CpG-ODN stimulation. IgM^high^CD23^low^ B cells were selected to represent overall innate-like B cell population based on the previous reports [19, 27], from which the four sub-types of innate-like B cells were identified by CD43 and CD93 labeling (Fig 3A). CD43 has been identified as a marker to define adaptive regulatory B cells from spleen MZ over their innate counterparts of B1 B cells in immune responses against bacterial infection [28]. CD93 has been used to discriminate between transitional 1 (T1) cells and mature B cells [29]. Addition of CpG-ODN to *E*. *coli* LPS or *P*. *gingivalis* LPS significantly reduced the percentage of CD43^-^CD93^-^ marginal zone (MZ) B cells (Fig 3B). Contrarily, the percentage of CD43^-^CD93^+^ transitional (T1) B cells was largely increased when treated with *E*. *coli* LPS or *P*. *gingivalis* LPS together with CpG-ODN (Fig 3C). The percentage of CD43^+^CD93^+^ innate response activator (IRA) B cells was increased by *E*. *coli* LPS or *P*. *gingivalis* LPS treatment alone, whereas addition of CpG-ODN to *E*. *coli* LPS or *P*. *gingivalis* LPS significantly reduced the percentage of IRA B cells (Fig 3D). The percentage of CD43^+^CD93^-^ B1 B cells was reduced only when treated with CpG-ODN together with *E*. *coli* LPS (Fig 3E).

### Suppression of Innate-like B cell apoptosis by *P*. *gingivalis* LPS and CpG-ODN

**Fig 4:**
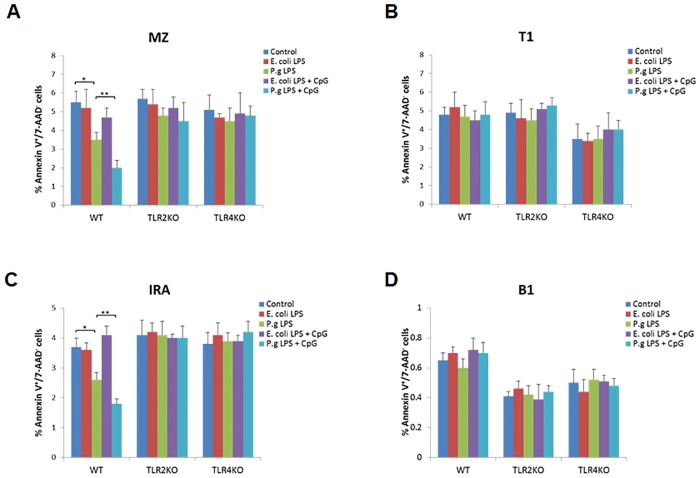
Early apoptosis analysis of innate-like B cell subsets after *E*. *coli* LPS, *P*. *gingivalis* LPS and CpG-ODN treatment. Splenocyte B cells were separated from WT, TLR2 KO and TLR4 KO mice and cultured 48 hours with *E*. *coli* LPS (10μg/ml), *P*. *gingivalis* LPS (10μg/ml), *E*. *coli* LPS (10μg/ml) + CpG (10μM), and *P*. *gingivalis* LPS (10μg/ml) + CpG (10μM). Cells were then stained by FITC-conjugated AnnexinV, 7-AAD, PE-conjugated anti IgM, PerCP-Cy5.5-conjugated anti CD23, APC-conjugated anti CD93, Pacific Blue-conjugated anti CD43 and measured by flow cytometry. In different innate-like B cell subsets including CD43^-^CD93^-^ marginal zone B cells (**A**), CD43^-^CD93^+^ transitional B cells (**B**), CD43^+^CD93^+^ innate response activator B cells (**C**) and CD43^+^CD93^-^ B1 B cells (**D**), the percentage of AnnexinV^+^/7-AAD^-^ (early apoptotic) B cells in different treatment groups of WT, TLR2 KO and TLR4 KO mice were analyzed and compared respectively (mean±SE, n = 5, *p<0.05, **p<0.01).

In order to determine the TLR-mediated regulation of innate-like B cell apoptosis, the percentage of AnnexinV^+^/7-AAD^-^ (early apoptotic) B cells from each innate-like B cell subpopulation was evaluated after treatment with LPS and CpG-ODN. In WT mice, *P*. *gingivalis* LPS but not *E*. *coli* LPS significantly inhibited the percentage of AnnexinV^+^/7-AAD^-^ B cells in MZ and IRA subpopulations (Fig 4A–4C), but not those in T1 or B1 subpopulations (Fig 4B–4D). In TLR2 KO or TLR4 KO mice, no differences were observed in the percentage of AnnexinV^+^/7-AAD^-^ B cells in each innate-like B cell subpopulation (Fig 4A–4D) after different treatments.

### Regulation of apoptosis-related genes in B cells by *P*. *gingivalis* LPS and CpG-ODN

**Fig 5:**
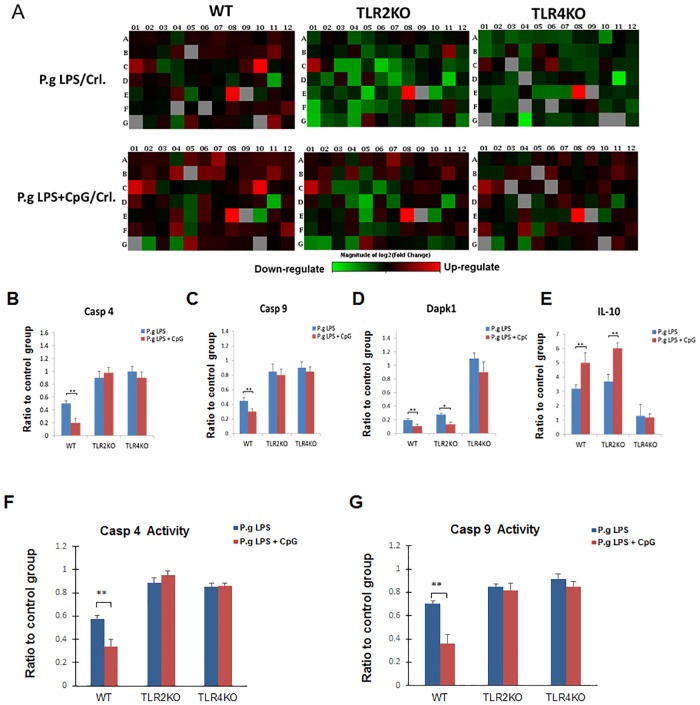
Differential mRNA levels of apoptosis-related genes in B cells after *P*. *gingivalis* LPS and CpG-ODN treatment. Splenocyte B cells were separated from WT, TLR2 KO and TLR4 KO mice and cultured 48 hours with *P*. *gingivalis* LPS (10μg/ml) and *P*. *gingivalis* LPS (10μg/ml) + CpG (10μM). Total RNA was extracted from these cells and used for RT² Profiler^™^ PCR Array Mouse Apoptosis (**A**). mRNA levels of Casp4 (**B**), Casp9 (**C**), Dapk1 (**D**) and IL-10 (**E**) in different groups of WT, TLR2 KO and TLR4 KO mice were determined by real-time PCR and the ratio of each treatment group to control group were analyzed and compared respectively (mean±SE, n = 3, *p<0.05, **p<0.01). Total cell lysis were used to detect Casp4 activity (**F**) and Casp9 activity (**G**) using fluorometric Assay kits. The ratio of each treatment group to control group were analyzed and compared respectively (mean±SE, n = 3, *p<0.05, **p<0.01).

Gene Arrays were performed with RNA samples from spleen B cells isolated from WT, TLR2 KO and TLR4 KO mice and cultured 48 hours with *P*. *gingivalis* LPS (10μg/ml) and *P*. *gingivalis* LPS (10μg/ml) + CpG (10μM) (Fig 5A). Genes obtained from array results that were up-regulated or down-regulated by more than 2-fold relative to control were selected and individually verified with separate real-time PCR reactions using the same gene specific primers. The results showed that pro-apoptotic genes, Caspase 4 (Casp 4) and Caspase 9 (Casp 9) were down-regulated by *P*. *gingivalis* LPS in B cells from WT mice, but not B cells from TLR2 KO or TLR4 KO mice (Fig 5B and 5C). CpG-ODN further enhanced such down-regulation of Casp 4 and Casp 9 in B cells from WT mice (Fig 5B and 5C). Furthermore, pro-apoptotic genes, death-associated protein kinase 1 (Dapk1) was down-regulated by *P*. *gingivalis* LPS in B cells from WT and TLR2 KO mice, but not those from TLR4 KO mice (Fig 5D). However, anti-apoptotic gene interleukin 10 (IL-10) was up-regulated by *P*. *gingivalis* LPS in B cells from WT and TLR2 KO mice, but not from TLR4 KO mice (Fig 5E). The down-regulation of Dapk1 and up-regulation of IL-10 were further enhanced by addition of CpG-ODN in B cells from WT and TLR2 KO mice, but not B cells from TLR4 KO mice (Fig 5D and 5E). To further study the functional changes of pro-apoptotic genes, Casp4 and Casp9 proteins activities were investigated from total cell lysis of each group. Consistent with mRNA results (Fig 5B and 5C), Casp 4 and Casp 9 activities were down-regulated by *P*. *gingivalis* LPS in B cells from WT mice, but not from TLR2 KO or TLR4 KO mice (Fig 5F and 5G); CpG-ODN further enhanced such down-regulation of Casp 4 and Casp 9 activities in B cells from WT mice (Fig 5F and 5G).

## Discussion

B lymphocytes are the predominant cells in established and advanced periodontal lesions, contributing to the B cell-mediated immune defenses as well as periodontal pathogenesis. However, the role of TLR signaling on B cells during periodontal diseases is not fully understood. In the present study, we have determined whether innate-like B cell apoptosis could be regulated by TLR ligands from periodontal pathogens.

In this study we elected to use high concentration of LPS in our cell culture experiments to test the effect of *P*. *gingivalis* LPS and *E*. *coli* LPS on B cell proliferation and apoptosis. We have previously tested extensively the dose response of cultured purified B cells to both *P*. *gingivalis* LPS and *E*. *coli* LPS in WT, TLR2 KO and TLR4 KO mice. Our results indicated that higher concentration of LPS (10ug/mL), especially *P*. *gingivalis* LPS, is needed to effectively stimulate purified B cell responses in the absence of other cells (S1 Fig). The purified B cells do not respond well to LPS probably due to the lacking of T cell help [30] and B cells from TLR2 and TLR4 KO mice respond poorly to LPS due to their deficiency in TLR2/4 signaling. Moreover, *P*. *gingivalis* LPS showed less induction on proliferation and stronger inhibition on apoptosis than *E*. *coli* LPS, suggesting a complexity of links between cell proliferation and apoptosis in B cells. It has been showed that cell proliferation and apoptosis may address both positive relationship [31, 32] and negative relationship [33] due to cell type, cellular environment and genetic background [34, 35] and further study is needed to investigate the links between proliferation and apoptosis in B cells.

We demonstrated that *P*. *gingivalis* LPS- or *E*. *coli* LPS-induced B cell proliferation was enhanced by CpG-ODN. However, B cell proliferation was differentially regulated by *P*. *gingivalis* LPS as compared to *E*. *coli* LPS. LPS derived from the periodontal pathogen *P*. *gingivalis* has been shown to differ from *E*. *coli* LPS in structure and function; therefore, triggering different intracellular inflammatory signaling pathways [36]. Studies have suggested that *P*. *gingivalis* LPS and *E*. *coli* LPS differently regulate cytokine production in human gingival fibroblasts [37]. *E*. *coli* LPS, but not *P*. *gingivalis* LPS stimulates IL-6 production of periodontal ligament cell [38]. Furthermore, the tetra- and penta-acylated lipid A structures of *P*. *gingivalis* LPS differentially activate TLR4-mediated NF-kappa B signaling pathway, and significantly modulate the expression of IL-6 and IL-8 in human gingival fibroblasts [39]. Our results indicated that *P*. *gingivalis* LPS, but not *E*. *coli* LPS suppressed the early and late apoptosis of B cells, which could be enhanced by CpG-ODN (Fig 2). It has long been recognized that stimulatory CpG-ODN has anti-apoptotic effect on B cells [40–42], indicating that CpG can act independently against cell apoptosis. However, our results showed that CpG-ODN and LPS induced anti-apoptotic effects involve common TLR signaling pathways (TLR2/4). Addition of CpG further enhanced gene expression profiles observed in LPS-treated group in WT but not TLR2/4 KO mice (Fig 5). This suggests that CpG-ODN induced enhancement of anti-apoptotic effect could be achieved through both LPS-dependent and independent mechanisms, which will be important to be addressed in future studies.

Recent studies have shown that *P*. *gingivalis* could manipulate TLR signaling and subvert leukocytes to create a favorable environment for a select community of bacteria that, in turn, adversely affects the periodontal tissues [43, 44]. Thus, this TLR ligands- induced dysregulation of apoptosis in B cells may cause autoimmune manifestations.

Our findings indicated that MZ B cells and IRA B cells were the predominant innate-like B cell subsets that their spontaneous programmed death was suppressed by *P*. *gingivalis* LPS and CpG-ODN. MZ B cell subset is critical for antibody-mediated protection against bacterial and viral infections at relatively early stages of infection [45]. Compared with follicular (FO) B cells, MZ B cells are more readily activated upon TLR stimulation [46]. These properties enable MZ B cells with an important role in host defense at the early stages of an innate immune response as well as adaptive immune response [19, 47]. IRA B cells are a recently identified effector B cell population that is functionally distinctive from B1a B cells and protects against microbial sepsis [18]. While sustained innate response can be protective [48] as well as pathogenic [49], further *in vivo* investigations are needed to determine whether disruption of B cell apoptosis could be another mechanism for *Porphyromonas gingivalis* to uncouple bacterial clearance from inflammation.

Casp 4 and Casp 9 are protease enzymes playing essential roles in programmed cell death (including apoptosis, pyroptosis and necroptosis) and inflammation [50, 51]. *P*. *gingivalis* LPS and *P*. *gingivalis* LPS + CpG-ODN significantly decreased the mRNA of Casp 4 and Casp 9 in B cells of WT mice but not of TLR2 KO and TLR4 KO mice, suggesting the inhibition of B cell apoptosis by *P*. *gingivalis* LPS and CpG-ODN was depended on both TLR2 and TLR4. However, Dapk1, a positive mediator of gamma-interferon induced programmed cell death [52], showed similar reduction in TLR2 KO mice not TLR4 KO mice compared with WT mice after *P*. *gingivalis* LPS and *P*. *gingivalis* LPS + CpG-ODN treatment. These results suggest that TLR4, but not TLR2, is essential to regulate Dapk1 in B cells by stimulation with *P*. *gingivalis* LPS and CpG-ODN. Moreover, up-regulation of IL-10 was also in TLR4-dependent manner. Thus, TLR2 and TLR4 signaling were differentially involved in regulating Casp 4/Casp 9 and Dapk1/IL-10 and their underlying mechanisms need to be further investigated.

In summary, our results provided new information about multiple TLR signaling on the control of innate-like B cell-apoptosis and may contribute to the development of therapeutic strategies that are effective in preventing and/or reducing periodontal disease pathogenesis.

## Supporting Information

S1 Fig B cell proliferative response to *P*. *gingivalis* LPS and *E*. *coli LPS stimulation*. *Purified B cells* (2×10^5^/well) were cultured in 200μl complete medium in 96-well plate for 2 days in the presence of *P*. *gingivalis* LPS or *E*. *coli LPS* (200ng, 2μg and 10μg/ml). MTS reagent was added (40μl/well) 4 hours before the termination of the experiment using a CellTiter 96 AQueous Assay kit (Promega Corp). After 4 hour incubation, the plate was read at OD 490nm using a microplate reader (BioTek). The absorbance of the formazan at 490nm was measured as an indication of cell proliferation. N = 3.
(PDF)
