# Supplementary material for: Lipopolysaccharides-Induced Suppression of Innate-Like B Cell Apoptosis Is Enhanced by CpG Oligodeoxynucleotide and Requires Toll-Like Receptors 2 and 4
Source: PLoS One. 2016 Nov 3;11(11):e0165862. doi: 10.1371/journal.pone.0165862 (PMC5094738; doi:10.1371/journal.pone.0165862)
Supplement: S1 Fig — Purified B cells (2×10^5^/well) were cultured in 200μl complete medium in 96-well plate for 2 days in the presence of P. gingivalis LPS or E. coli LPS (200ng, 2μg and 10μg/ml). MTS reagent was added (40μl/well) 4 hours before the termination of the experiment using a CellTiter 96 AQueous Assay kit (Promega Corp). After 4 hour incubation, the plate was read at OD 490nm using a microplate reader (BioTek). The absorbance of the formazan at 490nm was measured as an indication of cell proliferation. N = 3. (PDF) [file pone.0165862.s001.pdf]

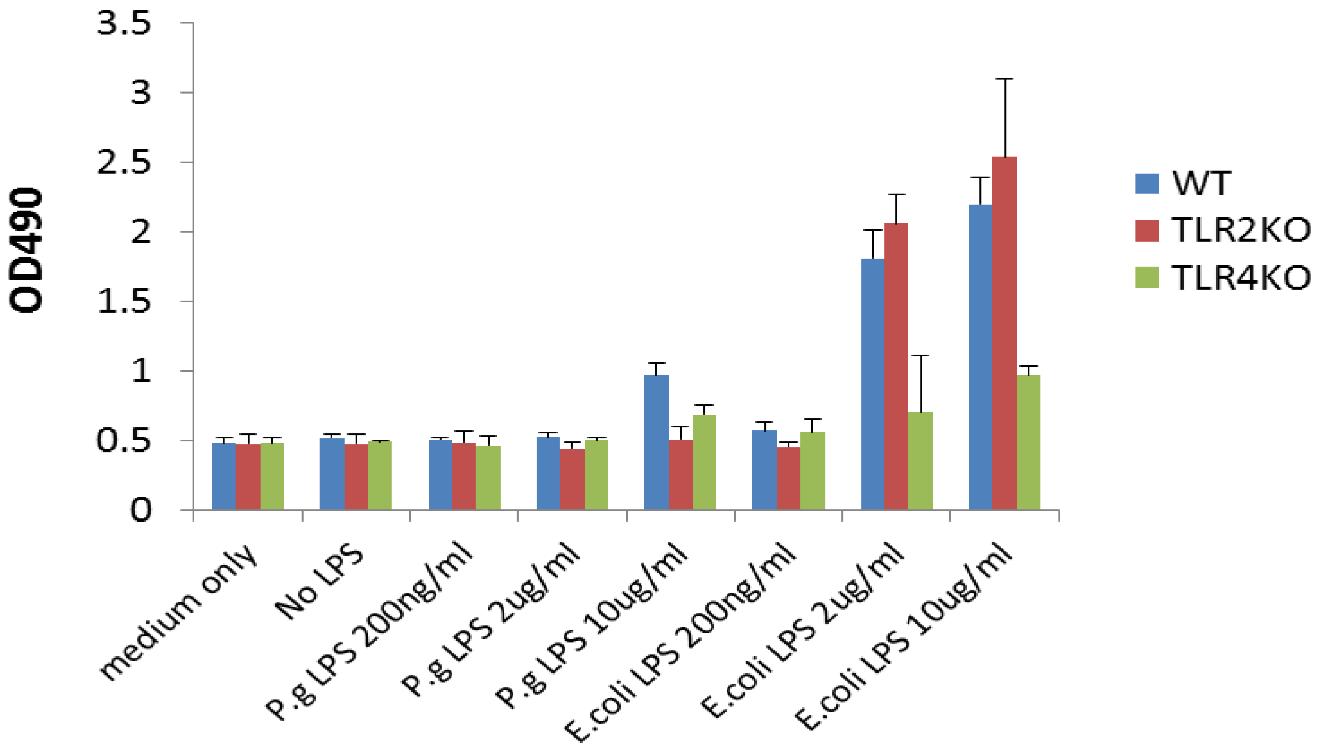

S1 Fig. B cell proliferative response to *P. gingivalis* LPS and *E. coli LPS stimulation.* *Purified B cells* (2×10^5/well) were cultured in 200µl complete medium in 96-well plate for 2 days in the presence of *P. gingivalis* LPS or *E. coli LPS* (200ng, 2µg and 10µg/ml). MTS reagent was added (40µl/well) 4 hours before the termination of the experiment using a CellTiter 96 AQueous Assay kit (Promega Corp). After 4 hour incubation, the plate was read at OD 490nm using a microplate reader (BioTek). The absorbance of the formazan at 490nm was measured as an indication of cell proliferation. N=3.
